# Modeling the impact of reproductive mode on masting

**DOI:** 10.1002/ece3.3214

**Published:** 2017-07-03

**Authors:** Yongjie Liu, Zhixia Ying, Shichang Wang, Jinbao Liao, Hui Lu, Liang Ma, Zhenqing Li

**Affiliations:** ^1^ State Key Laboratory of Vegetation and Environmental Change Institute of Botany Chinese Academy of Sciences Beijing China; ^2^ Department of Biology Centre of Excellence Plant and Ecosystem University of Antwerp Wilrijk Belgium; ^3^ College of Life Science Key Laboratory of Poyang Lake Environment and Resource Utilization Ministry of Education Nanchang University Nanchang China; ^4^ Key Laboratory of Animal Ecology and Conservation Biology Centre for Computational Biology and Evolution Institute of Zoology Chinese Academy of Sciences Beijing China; ^5^ Ministry of Education's Key Laboratory of Poyang Lake Wetland and Watershed Research Jiangxi Normal University Nanchang China; ^6^ University of Chinese Academy of Sciences Beijing China

**Keywords:** asexual reproduction, refined resource budget model, sexual reproduction, trade‐off

## Abstract

Masting is defined as the intermittent highly variable production of seed in a plant population. According to reproductive modes, that is, sexual and asexual reproduction, masting species can be separated into three groups, that is, (1) species, for example, bamboo, flower only once before they die; (2) species, for example, *Fagus*, reproduce sexually; and (3) species, for example, *Stipa tenacissima*, reproduce both sexually and asexually. Several theories have been proposed to explore the underlying mechanisms of masting. However, to our knowledge, no theory has been found to explain the mechanism of masting species that reproduce both sexually and asexually. Here we refine the Resource Budget Model by considering a trade‐off between sexual and asexual reproduction. Besides the depletion efficient (i.e., the ratio of the cost of seed setting and the cost of flowering), other factors, such as the annual remaining resource (i.e., the rest of the resource from the photosynthetic activity after allocating to growth and maintenance), the trade‐off between sexual and asexual reproduction, and the reproductive thresholds, also affect masting. Moreover, two potential reproductive strategies are found to explain the mechanisms: (1) When the annual remaining resource is relatively low, plants reproduce asexually and a part of the resource is accumulated as the cost of asexual reproduction is less than the annual remaining resource. Plants flower and set fruits once the accumulated resource exceeds the threshold of sexual reproduction; (2) when the annual remaining resource is relatively high, and the accumulated resource surpasses the threshold of sexual reproduction, masting occurs. Remarkably, under certain depletion efficient, more investigation in sexual reproduction will lead plants to reproduce periodically. Additionally, plants investigate less resource to reproduce periodically when depletion efficient keeps increasing as plants can reproduce efficiently. Overall, our study provides new insights into the interpretation of masting, especially for species that reproduce both sexually and asexually.

## 1 INTRODUCTION

Masting or mast seeding is a synchronous, highly variable reproduction of perennial plants (Janzen, 1971; Kelly, 1994; Kelly & Sork, 2002). As one of the classical topics in ecology, it has attracted a lot of attention in the literature. In general, many masting species have been found, and these species can be separated into three groups according to their reproductive modes. First group includes species, for example, bamboo (Janzen, 1976), that flower once before they die. Second group contains species only with sexual reproduction, for example, *Fagu*s (Fietz, Kager, & Schauer, 2009; Nilsson, 1985; Yasaka, Terazawa, Koyama, & Kon, 2003), *Pinus* (Climent et al., 2008; Mooney, Linhart, & Snyder, 2011), *Quercus* (Sork, 1993; Sork & Bramble, 1993; Yi and Liu, 2014; Yi, Wang, Liu, & Zhang, 2015), *Podocarpus* (Andrew, Neal, & Philip, 2016), *Strobilanthes* (Tsvuura, Griffiths, Gunton, & Lawes, 2011), *Corylus* (Yang, Liu, Liu, & Yi, 2014), and *Chionochloa* (Rees, Kelly, & Bjørnstad, 2002). Species, for example, *Stipa tenacissima* L. (Haase, Pugnaire, & Incoll, 1995), which reproduces both sexually and asexually, belongs to the third group. Most of the above‐mentioned studies dealt with the first two groups of masting species. However, no studies have to our knowledge explored the underlying mechanism of masting species in the third group.

Many hypotheses have been proposed to explain the proximate and ultimate causes of masting (Kelly, 1994; Kelly & Sork, 2002). One of the proximate explanations is resource‐matching hypothesis, proposing that seeds may vary with resources availability (Houle & Filion, 1993). Another is the environmental prediction hypothesis emphasizing that plants can predict which year will be the best time for seedling, so plants will seed in the right year (Smith, Hamrick, & Kramer, 1990). Subsequently, researchers explored the ultimate cause—selective advantages of masting, which is known as the economy of scale. One of them is pollination efficiency hypothesis, suggesting that large flowering efforts (in mast years) increase the chance of successful pollination (Koenig, Mumme, Carmen, & Stanback, 1994). Another is predator satiation hypothesis proposing that high interannual variability of seeds limits the population density of predators by starving them in low seed years and satisficing them in high seed year, and this allows a portion of seeds to escape (Janzen, 1971, 1976; Silvertown, 1980). Moreover, dispersal hypothesis assumes that seed dispersal or dispersal distance will be enhanced in mast year due to a large number of seeds eaten and then dispersed by animals. Isagi, Sugimura, Sumida, and Ito (1997) and Satake and Iwasa (2000) introduced a resource budget model (RBM) to explore the strategies of resource allocation at plant individual scale. It proposes the dynamics of internal energy reserves that allowing plants generate a large fluctuation of reproductive activity among years. Then, Satake and Iwasa (2002a,b) studied the effect of the fluctuating of reproductive threshold between years on the annual productivity of plants. Tachiki, Iwasa, and Satake (2010) further examined the mechanism of synchronous reproduction of different species when they shared common pollinators. However, none of the above‐mentioned theories has been used to explain the mechanism of species that can reproduce both sexually and asexually.

Until now, RBM has been thought to be one of the most powerful models to explain the underlying mechanism of masting and it has been confirmed theoretically or empirically at least for a few species (Crone, Miller, & Sala, 2009; Crone & Rapp, 2014). RBM did not include the effect of asexual reproduction on masting or it treated the asexual reproduction as a part of plant growth. However, numerous studies have found a trade‐off in resource allocation between sexual and asexual reproduction (Fu, Wang, Liu, Nijs, & Li, 2010; Klimes, Klimesova, Hendriks, & Van Groenendael, 1997; Liao, Li, Hiebeler, Iwasa, et al., 2013; Liao, Li, Hiebeler, El‐Bana, et al., 2013; Weppler, Stoll, & Stöcklin, 2006; Xiao, Dong, Wang, & Lan, 2016; Zhang & Zhang, 2006). Moreover, the resource limitation hypothesis stated that allocation to sexual reproduction was expected to reduce the fraction of energy assigned to asexual reproduction at the same time (Coelho, Deboni, & Lopes, 2005; Olejniczak, 2001). Accordingly, the trade‐off in resource allocation between two reproductive modes might affect masting for species with both sexual and asexual reproduction. Until now, no studies have considered or even attempted to explore the effect of this trade‐off on masting. Hence, we extend RBM by considering the resource allocation between different reproductive modes and explore its effects on masting. Specially, we first modify the RBM to build the refined resource budget model (RRBM) by taking the trade‐off in resource allocation between sexual and asexual reproduction into account. Then, we analyze the stability of RRBM theoretically and conduct simulations to explore the effect of resource allocation between sexual and asexual reproduction on the seeding of plant individuals. Finally, we discuss the limits of RRBM and provide the suggestions for further studies.

## 2 MODEL DESCRIPTIONS

An adult plant individual is assumed to have a constant photosynthetic activity each year. A part of the resource from the photosynthetic activity is allocated to growth and maintenance, while the annual remaining resource (*P*_s_) is stored in the plant (e.g., root, branch, and trunk) and accumulates until it is enough for reproduction. Here *y*(*t*) is assigned to be the accumulated resource of an adult plant individual at the beginning of year *t*. Generally, plants accumulate resource and then reproduce both sexually and asexually, and they tend to allocate resource to asexual reproduction under severe environmental conditions (Barrett, 2015; Zhang, Zhang, & Barrett, 2010). It suggests that, to some extent, asexual reproduction requires less resource than sexual reproduction. So the threshold of asexual reproduction (*L*_1_) is assumed to be lower than the threshold of sexual reproduction (*L*_2_) (*L*_1_ < *L*_2_). Plants will reproduce as one of the following three cases, that is, resource accumulation (RA), asexual reproduction (AR), or both sexual and asexual reproduction (SAR). Specifically, (1) RA: If the accumulated resource of a plant in year *t* is less than the asexual reproduction threshold (*L*_1_) (i.e., *y*(*t*) + *P*_s_ < *L*_1_), the plant will not reproduce but accumulate resource until the accumulated resource is enough for reproduction; (2) AR: If the accumulated resource in year *t* exceeds the asexual reproduction threshold (*L*_1_) but not surpasses the sexual reproduction threshold (*L*_2_) (i.e., *L*_1_ < *y*(*t*) + *P*_s_ < *L*_2_), the plant will reproduce asexually. The cost of asexual reproduction is *d* * (*y*(*t*) + *P*_s_ − *L*_1_), where *d* refers to the ratio of resource allocated to asexual reproduction. Hence, the annual remaining resource of the plant is *y*(*t*) + *P*_s_ − *d* * (*y*(*t*) + *P*_s_ − *L*_1_); (3) SAR: If the accumulated resource in year *t* is more than sexual reproduction threshold (*L*_2_) (i.e. *y*(*t*) + *P*_s_ > *L*_2_), the plant will reproduce both sexually and asexually. Here, the resource cost of flowering is *C*_f_ = *p* * (*y*(*t*) + *P*_s_ − *L*_1_), where *p* indicates the ratio of resource allocated to sexual reproduction. In other words, *p* is used to measure the trade‐off of resource between sexual and asexual reproduction. Accordingly, the cost of asexual reproduction is *C*_v_ = *q* * (*y*(*t*) + *P*_s_ − *L*_1_), where *q* is assigned to measure the resource that allocated to asexual reproduction. Some flowers can be pollinated after flowering and then set seeds. Here, the cost of seed setting is represented by *C*_a_. Given that the cost of seed setting is positively proportional to the cost of flowers, so *C*_a_ can be presented as *C*_a_ = *R* * *C*_f_ = *R* * *p* * (*y*(*t*) + *P*_s_ − *L*_1_), where *R* is the depletion coefficient. Hence, the total reproduction cost is *C*_f_ + *C*_a_ + *C*_v_. After reproduction, the accumulated resource of the plant in the year (*t* + 1) is *y*(*t*) + *P*_s_ − *C*_f_ − *C*_a_ − *C*_v_ = *y*(*t*) − (*p* + *q* + *R* * *p*) * (*y*(*t*) + *P*_s_ − *L*_1_) + *P*_s_.

Accordingly, the accumulated resource of the plant at the beginning of year (*t* + 1) can be briefly expressed as follows:

(1) $$y\left(t+1\right)=\left\{\begin{array}{cc} y\left(t\right)+P_{s}, & ify\left(t\right)+P_{s}<L_{1}\\ y\left(t\right)-d*\left(y\left(t\right)+P_{s}-L_{1}\right)+P_{s}, & ifL_{1}\leq y\left(t\right)+P_{s}\leq L_{2}\\ y\left(t\right)-\left(p+q+R*p\right)*\left(y\left(t\right)+P_{s}-L_{1}\right)+P_{s}, & ify\left(t\right)+P_{s}>L_{2} \end{array}\right.$$

Equation (1) is a modified version of resource budget model. In the following sections, without special declare this model is referred as the RRBM. All the parameters used in RRBM are shown in Table 1.

**Table 1:** Parameters and their values used in refined resource budget model

Parameter	Definition	Values
*y*(*t*)	Accumulated resource at year *t*	
*L*_1_	Threshold of asexual reproduction	4.0
*L*_2_	Threshold of sexual reproduction	6.0
*P*_s_	Remaining resource after growth and maintenance	3.0
*p*	Flowering coefficient, resource for sexual reproduction	(0, 1)
*q*	The asexual reproductive coefficient	(0, 1)
*d*	The degree of resource depleted by asexual reproduction	(0, 1)
*R*	Depletion coefficient	

## 3 RESULTS

Here, we first analyze the stability of RRBM by exploring its fixed points via stability analysis and then conduct simulations with RRBM as the stability analysis cannot enable us to separate the chaos and the periodic cycles as these simulations are very useful to understand the dynamics of this model. Masting emerges when the number of seeds shows synchronous and highly variable traits.

### 3.1 Theoretical analysis and numerical simulation

To achieve the fixed points in Equation (1), *y*(*t *+ 1) is assumed to equal to *y*(*t*). Two fixed points are found (Table 2). The first one is *y*_1_* (*y*_1_* = *L*_1_ − *P*_s_ + *P*_s_/(*p* + *q* + *p* * *R*)) when the accumulated resource is above the threshold of sexual reproduction (*y*(*t*) + *P*_s_ > *L*_2_). The existent condition is *P*_s_ > (*p* + *q* + *R* * *p*) * (*L*_2_ − *L*_1_). It is necessary to mention that Lyapunov exponent is used to distinguish chaotic behaviour from equilibrium and stable periodic cycles (Ruelle 1990). It is negative if the trajectory converges to either equilibrium or a stable periodic cycle, while it is positive when the trajectories tend to deviate from each other (Satake & Iwasa, 2000). In this case, Lyapunov exponent is λ = ln|1−*p* − *q* − *p* * *R*|. Accordingly, when *p* * *R* < 2 − *p* − *q* the fixed point *y*_1_* is locally stable, which means the plant has a constant amount of accumulated resource and a constant reproductive pattern in the following years. The second fixed point is *y*_2_* (*y*_2_* = *L*_1_ + *P*_s_/*d* − *P*_s_) when the accumulated resource is higher than the threshold of asexual reproduction but is less than the threshold of sexual reproduction (*L*_1_ ≤ *y*(*t*) + *P*_s_ ≤ *L*_2_). The existent condition of this fixed point is *P*_s_ < (*L*_2_ − *L*_1_) * *d*. Here, Lyapunov exponent is λ = ln|1 − *d*|. It is always negative since 0 < *d* < 1. So the fixed point *y*_2_* is locally stable if it exists. Accordingly, the reproductive dynamics of the plant is summarized (Table 3), and they are explained in details as follows:

1. If *P*_s_ < (*L*_2_ − *L*_1_) * *d*, that is, the annual remaining resource (*P*_s_) < the minimal resource consumption at AR stage, the fixed point *y*_2_* = *L*_1_ + *P*_s_/*d* − *P*_s_ exists and is locally stable. The cost of flowering (*C*_f_) and the cost of setting seeds (*C*_a_) are zero (Fig. 1a). It means that the plant tends to reproduce asexually rather than sexually when the annual remaining resource (*P*_s_) is smaller than the minimal cost of asexual reproduction. It makes sense in terms of evolution, as asexual reproduction is the only choice for the plant when the sexual reproduction is limited. This also indicates that the plant can keep a constant accumulated resource for reproduction every year through reproducing asexually under the condition of (*P*_s_ < (*L*_2_ − *L*_1_) * *d*).

**Figure 1:**
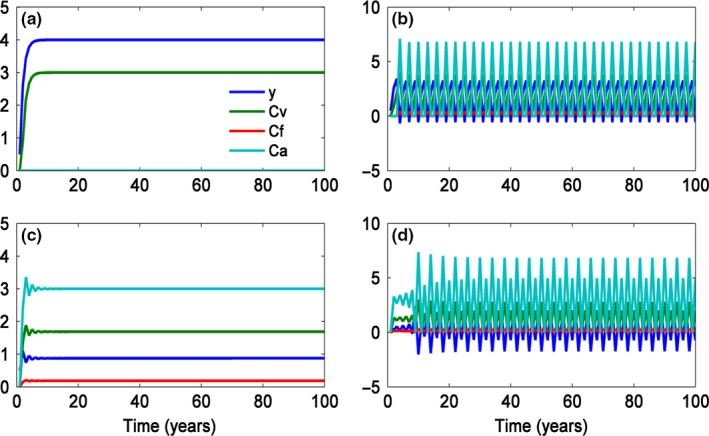
Dynamics of sexual and asexual reproduction of a plant with RRBM. (a) The plant only reproduces asexually, *L*_1_ = 2; *P*_S_ = 3; *L*_2_ = 8; *d* = 0.6; *p* = 0.1; *q* = 0.9 and *R* = 6. (b) The plant reproduces both sexually and asexually, *L*_1_ = 2; *P*_S_ = 3; *L*_2_ = 6; *d* = 0.6; *p* = 0.1; *q* = 0.9 and *R* = 6. (c) The plant reproduces both sexually and asexually in a constant way, *L*_1_ = 2; *P*_S_ = 3; *L*_2_ = 3; *d* = 0.6; *p* = 0.1; *q* = 0.9 and *R* = 6. (d) The plant individual reproduces both sexually and sexually, but in a unstable way, *L*_1_ = 2; *P*_S_ = 3; *L*_2_ = 3; *d* = 0.6; *p* = 0.1; *q* = 0.9 and *R* = 12
2. If (*p* + *q* + *R* * *p*) * (*L*_2_ − *L*_1_) > *P*_s_ > (*L*_2_ − *L*_1_) * *d*, that is, the maximal resource consumption at SAR stage > the accumulated resource for reproduction (*P*_s_) > the minimal resource consumption at AR stage. No fixed point exists under this condition. In other words, it is difficult for the plant to reproduce sexually under the condition of (*p* + *q* + *R* * *p*) * (*L*_2_ − *L*_1_) > *P*_s_. This also indicates that the annual remaining resource (*P*_s_) is relatively low that cannot compensate the cost of sexual reproduction. While the plant can reproduce asexually and thus accumulate the rest resource under the condition of *P*_s_ > (*L*_2_ − *L*_1_) * *d*. So a part of the resource can be reserved during this process, plants can reproduce sexually after several years when the resource is above the threshold of sexual reproduction. It suggests that plants can reproduce asexually in some years and accumulate resource during those years and then reproduce sexually when the resource is above the reproductive threshold. For example, Fig. 1b shows the accumulated resource at year *t* (*y*(*t*)), asexual reproduction cost (*C*_v_), flowering cost (*C*_f_), and the cost of setting seeds (*C*_a_) variy periodically. This implies that the plant will reproduce asexually and sexually through a variable pattern under the condition of ((*p* + *q* + *R* * *p*) * (*L*_2_ − *L*_1_) > *P*_s_ > (*L*_2_ − *L*_1_) * *d*).
3. If (*p* + *q* + *R* * *p*) * (*L*_2_ − *L*_1_) < *P*_s_ and *R* * *p* < 2 − *p* − *q*, that is, the maximal cost of reproduction including sexual and asexual reproduction is less than the annual remaining resource, plants will have the minimal cost of setting seeds (min(*C*_a_) = R*p*(*L*_2_ − *L*_1_))), which can make plants avoid losing too much resource and blocking the sexual reproduction in the next year. The fixed point *y*_1_* = *L*_1_−*P*_s_ +*P*_s_/(1 + *p* * *R*) exists, and it is locally stable (Fig. 1c). This also suggests that if the cost of flowering (1/*d* > (*L*_2_ − *L*_1_)/*P*_s_) is small, the cost of setting seeds will be also small ((*L*_2_ − *L*_1_)/*P*_s_ > 1/(1 + *R* * *p*)). As a result, plants will reproduce sexually and asexually.
4. If (*L*_2_ − *L*_1_) * (*p* + *q* + *R* * *p*) < *P*_s_ and *R* * *p* > 2 − *p* − q, that is, the maximal cost of reproduction (sexual and asexual reproduction) is less than the annual remaining resource, but the cost of setting seeds is large enough to block the sexual reproduction in the next year; then, the fixed point *y*_1_* = *L*_1_ − *P*_s_ + *P*_s_/(1 + *p* * *R*) exists, but it is unstable (Fig. 1d). It means that the cost of seed setting is too much, which makes the accumulated resource to be lower than the threshold of reproduction. Accordingly, the plant will set seed in a variable way.

**Table 2:** Two fixed points and their existent conditions in refined resource budget model

Scene	Fixed points	Existent conditions
(1) *y*(*t*) > *L*_2_	$y_{1}^{*}=L_{1}-P_{s}+P_{s}/\left(p+q+p*R\right)$	*P*_s_ > (*p* + *q* + *R* * *p*) * (*L*_2_ − *L*_1_)
(2) *L*_1_ ≤ *y*(*t*) ≤ *L*_2_	$y_{2}^{*}=L_{1}+P_{s}/d-P_{s}$	*P*_s_ < (*L*_2_ − *L*_1_) * *d*

**Table 3:** Reproductive dynamics of plants with stable analysis of refined resource budget model, where four scenes are classified according to the two fixed points

Scene	Cases	Criteria
(1)	*y*_1_* is absent; *y*_2_* exists and is stable	*P*_s_ < (*L*_2_−*L*_1_)*d
(2)	*y*_1_* is absent; *y*_2_* is absent	(*p* + *q* + *R* * *p*) * (*L*_2_−*L*_1_) > *P*_s_ > (*L*_2_−*L*_1_) * *d*
(3)	*y*_1_* exists and is stable; *y*_2_* is absent	*P*_s_ > (*p* + *q* + *R* * *p*) * (*L*_2_−*L*_1_) and *R* * *p* < 2 − *p* − *q*
(4)	*y*_1_* exists but is unstable; *y*_2_* is absent	*P*_s_ > (*p* + *q* + *R* * *p*) * (*L*_2_ − *L*_1_) and *R* * *p* > 2−*p*−*q*

### 3.2 Effects of sexual and asexual reproduction and depletion coefficient on masting

**Figure 2:**
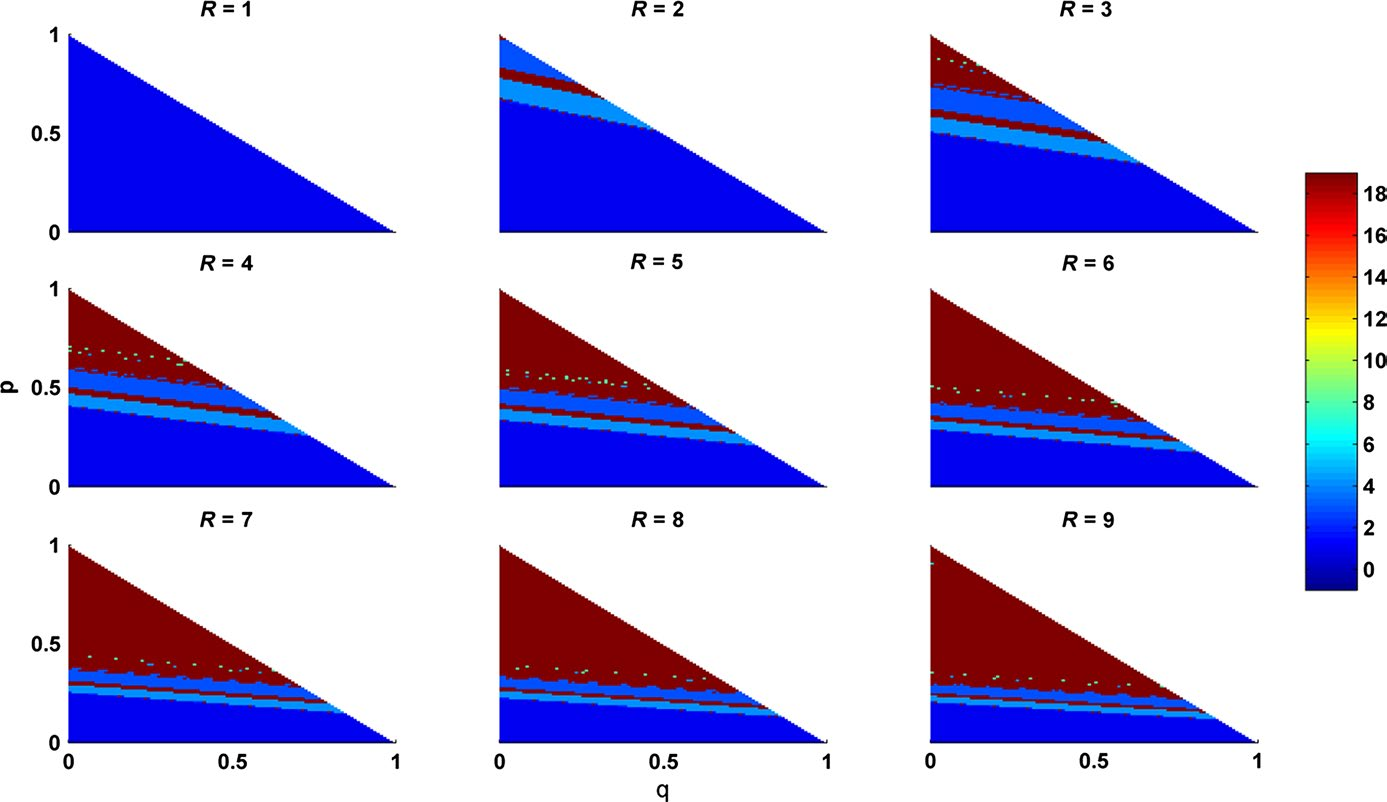
Simulations of the effects of sexual reproduction (flowering coefficient (*p*)) and asexual reproduction (*q*) on masting with varying depletion coefficient (*R*) in RRBM. The color bar shows the reproductive period: −1 means plants reproduce asexually every year; 0 means plants reproduce sexually every year; and 1–18 means plants reproduce sexually every 1–18 years; while 19 means plants reproduce either with a reproductive period large than 18 or with no regular reproductive period. Other parameters are *L*_1_ = 2; *P*_S_ = 3; *L*_2_ = 3 and *d* = 0.6

In order to explore the effects of asexual reproductive coefficient (*q*), depletion coefficient (*R*), and the flowering coefficient (*p*) on masting in RRBM, simulations are conducted by varying the values of *R* from 1 to 9. Moreover, to simulate the stable states of the plant individual, a series of simulations are conducted by varying the values of the flowering coefficient (*p*) and the asexual reproductive coefficient (*q*) under a certain value of *R* (Fig. 2). Results show that if the depletion coefficient (*R*) increases, plants tend to reproduce with a longer or irregular reproductive period. As shown in Fig. 2, the red area enlarges with the increasing of the value of *R*. Specifically, if *R* is small (e.g., *R* = 1), plants reproduce constantly every year, and varying *p* and *q* will not change the result. However, different reproductive periods appear when it increases (e.g., *R* = 2) on the condition of a relative large *p* (e.g., *R* > 0.5), which can be understood as more investigation in sexual reproduction will make plants to reproduce periodically. While if *R* keeps increasing (e.g., *R* = 3), plants can reproduce periodically by investigating less in sexual reproduction (i.e., even a small *p* can make the plant reproduce periodically).

## 4 DISCUSSION

Numerous studies of masting have been conducted, which vary from field experiments, modeling to observations. However, no studies have to our knowledge explored the underlying mechanism of masting species with both sexual and asexual reproduction, for example, *S. tenacissima* L. (Haase et al., 1995). We build the RRBM by considering the effect of reproductive modes on masting. In consistent with our expectation, four key factors, that is, depletion coefficient (*R*) and sexual reproduction (*p*), reproductive threshold (*L*), and the remaining resource (*P*_s_), affect masting in RRBM. The model, which refines RBM, provides new insights to explain the mechanism of masting.

Isagi et al. (1997) found that the main factor affected masting was the depletion coefficient *R* (i.e., the ratio of the cost of seed setting and the cost of flowering). Specifically, (1) species reproduced constantly when their depletion coefficient was small (*R* < 1); (2) while species reproduced irregularly when they had a large depletion coefficient (*R* ≥ 1). Moreover, the reproductive period enlarged and be unpredictable when the depletion coefficient increasing. These results are confirmed by our model (Fig. 2). Furthermore, we also find that the irregular reproduction is affected not only by depletion coefficient (*R*), but also by sexual reproduction (*p*), reproductive threshold (*L*), and the annual remaining resource (*P*_s_) (Fig. 2, Table 3). For example, masting appears when the aforementioned four factors fit the conditions mentioned in Section 3.1 (2) or (4). Generally, the trade‐off of reproductive modes indeed affects masting, and this trade‐off should be considered in the future study on masting.

Masting species benefit from the two reproductive modes, that is, sexual and asexual reproduction. On the one hand, species gets profit from asexual reproduction through the following three ways. One is that asexual reproduction enables plants to reproduce under condition of limited sexual reproduction (Barrett, 2015). Moreover, species benefits in exploring resource (e.g., light, nutrient) under rough environment or in dispersing from the unfavorable conditions. Furthermore, asexual reproduction costs less comparing with sexual reproduction. On the other hand, sexual reproduction of those species also benefit from the asexual production. Theoretical analyses show that species can save accumulated resource through asexual reproduction in the previous years or through sexual reproduction under a certain condition. Both of these two reproductive modes enable masting species to adapt to the environment. Consequently, the trade‐off between reproductive modes affects the plant seeding, which has been overlooked.

Simulations show that different masting species form different reproductive cycles. It should be noted that reproductive period or reproductive cycle means the average period of a long‐term recording as few stable reproductive cycle can be found in nature. For example, masting species with two‐year reproductive cycle, which is mostly found in species like apple, *Quercus stellata* and *Quercus velutina* (Sork, 1993); species that reproduce 3 year a cycle such as *Quercus imbricaria* (Sork, 1993) could be explained as either less resource is allocated to sexual reproduction or depletion coefficient increases; In Fig. 2, when *R* > 2, two chaotic areas (red) are found. Species, for example, more than 2 years of species *Pinus ponderosa* (Mooney et al., 2011), two‐ to three‐year cycle of species such as *Fagus Crenata* Blume (Abe et al., 2016), three‐ to five‐year cycle for *Quercus rubra* and four‐ to ten year cycle for *Quercus alba* (Liebhold, Elkinton, Williams, & Muzika, 2000; cited from Olson, 1974) and other Quercus (Sork, 1993); bamboo (e.g., more than 120‐year cycle) (Veller, Nowak, & Davis, 2015), may be fit in those conditions. But the underlying mechanisms are still unclear, further researches needed to be carried out.

Refined resource budget model is still a simplified theoretical model even it extends RBM. Assumptions and the values of parameters in RRBM should be tested with further field and experimental researches. For example, (1) the assumption of reproductive thresholds: Noble, Bell, and Harper (1979) stressed that it is critical to consider asexual reproduction when studying the reproductive allocation. Hence, asexual reproduction should be considered as a reproductive mode rather than as clonal growth. Moreover, few studies explored the reproductive thresholds, especially the threshold of asexual reproduction. Even those studies that conducted on reproductive thresholds found confusing results. Schmid, Bazzaz, and Weiner (1995) revealed that there may be a small threshold size for asexual reproduction that could not be detected. While other studies found that plants may reproduce asexually after flowering (Rautiainen, Koivula, & Hyvärinen, 2004) or plants may reproduce asexually and sexually at the same time (Mendez & Obeso, 1993). In RRBM, the threshold of asexual reproduction is assumed to be ahead of sexual reproduction (*L*_1_ < *L*_2_). However, these two cases, that is, asexual reproduction appears ahead of sexual reproduction (*L*_1_ > *L*_2_) and asexual and sexual reproduction emerge at the same time (*L*_1_ = *L*_2_), should also be explored in RRBM in the future. Therefore, more studies about the reproductive thresholds, we suggest, should be conducted, especially for species that can reproduce both sexually and asexually. (2) The assumption of the resource is as follows: Several studies have found that accumulated carbohydrate might not necessary in RBM (Hoch, Siegwolf, Keel, Körner, & Han, 2013; Ichie et al., 2013), while accumulating evidence shows that nitrogen or other nutrients play a crucial role in regulating of flowering (Crone et al., 2009; Fernández‐Martínez, Vicca, Janssens, Espelta, & Peñuelas, 2017; Han, Kabeya, Lio, Inagaki, & Kakubari, 2004; Miyazaki et al., 2014). This should be tested in RRBM even it might not change the mechanism in the model. In addition, as did in RBM (Isagi et al., 1997; Satake & Iwasa, 2000), RRBM did not consider the trade‐off of resource between growth and reproduction. While studies revealed that this tread‐off should be considered (Wenk & Falster, 2015) at least for species such as *Quercus ilex* (Koenig, Knops, Carmen, & Pearse, 2015) and *Fagus sylvatica* (Mund et al., 2010). (3) The assumption of environmental conditions is as follows: Reproductive thresholds are a species‐specific trait, but they are affected by (non‐)biotic factors (Martin, Piqué, Carevic, Fernández, & Alejano, 2015). For example, an exceptional cold winter can low the reproduce threshold (*L*_1_, *L*_2_). Plants have a chance to reproduce in the following year accordingly, which would not happen as the accumulated resource is less than the reproductive threshold under the normal condition (Pearse, Koenig, & Knops, 2013). Here, to simplify the RRBM, values of *P*_s_ and *L*_2_ are derived from the study of Isagi et al. (1997). Asexual reproduction threshold and the degree of resource depleted by asexual reproduction are set as constant values. (4) The potential effect of the phenology is as follows: Further studies on the setup time of internal resources are essential to discover the causes and consequences of masting. On the one hand, plants need sufficient flowers before the mast seeding year occurs. If every plant responses to resource availability and starts the process of flower initiation, it is crucial to focus on flower differentiation within the winter buds at the resource level. On the other hand, flower differentiation does not directly affect fruit development occurs a few months thereafter, although there may be an autocorrelation between the flower differentiation and the fruit development.

## AUTHOR CONTRIBUTIONS

Y.L. and Z.L. designed the research. Y.L. and Z.Y. built the model and conducted the simulations. Y.L. and Z.L. wrote the manuscript. All the authors contributed to the interpretation of the results and to the text.
